# Measuring Electronic Health Literacy: Development, Validation, and Test of Measurement Invariance of a Revised German Version of the eHealth Literacy Scale

**DOI:** 10.2196/28252

**Published:** 2022-02-02

**Authors:** Matthias Marsall, Gerrit Engelmann, Eva-Maria Skoda, Martin Teufel, Alexander Bäuerle

**Affiliations:** 1 Clinic for Psychosomatic Medicine and Psychotherapy LVR–University Hospital Essen University of Duisburg-Essen Essen Germany; 2 Institute for Patient Safety University Hospital Bonn Bonn Germany

**Keywords:** eHealth, eHeals, health literacy, factor analysis, validation, measurement invariance, internet, health information

## Abstract

**Background:**

The World Wide Web has become an essential source of health information. Nevertheless, the amount and quality of information provided may lead to information overload. Therefore, people need certain skills to search for, identify, and evaluate information from the internet. In the context of health information, these competencies are summarized as the construct of eHealth literacy. Previous research has highlighted the relevance of eHealth literacy in terms of health-related outcomes. However, the existing instrument assessing eHealth literacy in the German language reveals methodological limitations regarding test development and validation. The development and validation of a revised scale for this important construct is highly relevant.

**Objective:**

The objective of this study was the development and validation of a revised German eHealth literacy scale. In particular, this study aimed to focus on high methodological and psychometric standards to provide a valid and reliable instrument for measuring eHealth literacy in the German language.

**Methods:**

Two internationally validated instruments were merged to cover a wide scope of the construct of eHealth literacy and create a revised eHealth literacy scale. Translation into the German language followed scientific guidelines and recommendations to ensure content validity. Data from German-speaking people (n=470) were collected in a convenience sample from October to November 2020. Validation was performed by factor analyses. Further, correlations were performed to examine convergent, discriminant, and criterion validity. Additionally, analyses of measurement invariance of gender, age, and educational level were conducted.

**Results:**

Analyses revealed a 2-factorial model of eHealth literacy. By item-reduction, the 2 factors information seeking and information appraisal were measured with 8 items reaching acceptable-to-good model fits (comparative fit index [CFI]: 0.942, Tucker Lewis index [TLI]: 0.915, root mean square error of approximation [RMSEA]: 0.127, and standardized root mean square residual [SRMR]: 0.055). Convergent validity was comprehensively confirmed by significant correlations of information seeking and information appraisal with health literacy, internet confidence, and internet anxiety. Discriminant and criterion validity were examined by correlation analyses with various scales and could partly be confirmed. Scalar level of measurement invariance for gender (CFI: 0.932, TLI: 0.923, RMSEA: 0.122, and SRMR: 0.068) and educational level (CFI: 0.937, TLI: 0.934, RMSEA: 0.112, and SRMR: 0.063) were confirmed. Measurement invariance of age was rejected.

**Conclusions:**

Following scientific guidelines for translation and test validation, we developed a revised German eHealth Literacy Scale (GR-eHEALS). Our factor analyses confirmed an acceptable-to-good model fit. Construct validation in terms of convergent, discriminant, and criterion validity could mainly be confirmed. Our findings provide evidence for measurement invariance of the instrument regarding gender and educational level. The newly revised GR-eHEALS questionnaire represents a valid instrument to measure the important health-related construct eHealth literacy.

## Introduction

### Background

The concept of health literacy emerged in the 1990s as a competence to gather health information and use it to address health questions and problems [1]. Nutbeam [2] defined health literacy as “cognitive and social skills which determine the motivation and ability of individuals to gain access to, understand, and use information in ways which promote and maintain good health.” In the following years, health literacy has turned out to be an important predictor for various health outcomes (eg, behavior of patients with diabetes mellitus or heart failure) [3,4]. The World Health Organization has declared health literacy as a key determinant of health and defined it as a Sustainable Development Goal [5].

With the rise of the internet as a source of information, the gathering of health information was no longer limited to professional or face-to-face health sources but was available from many different health topic websites [6]. With the increasing availability of health information on the internet, the number of people using this source for seeking health information rose as well [7,8]. However, sources on the internet contain inconsistent information as contributions are not by professionals only [9]. As a result, the amount and differences in quality of information provided on the internet may lead to health information overload [10]. For example, in 2020, COVID-19 became a global pandemic, and disease-related information, especially from the internet, grew exponentially, leading to an “infodemic” [11,12]. Not only is a large amount of information available, but a significant amount of it must be considered misinformation because the sources of the information must be classified questionable [13,14].

For the context of information from the internet, Norman and Skinner [15] applied the concept of health literacy to electronic health literacy (eHealth literacy). With the development of the eHealth Literacy Scale (eHEALS) questionnaire [16], the concept of eHealth literacy became measurable and emerged as a growing interest in psychological and medical health sciences. Systematic reviews have shown that eHEALS is associated with different health-related outcomes, but findings could not be consistently confirmed [17,18]. Associations of eHealth literacy with different health outcomes have been found, such as health intentions [19], acquiring health knowledge [20-23], and health prevention behavior [21,24,25]. Furthermore, research showed associations between eHealth literacy and healthy behaviors like exercise behavior, balanced nutrition, and regular breakfast [26,27]. In the context of COVID-19, associations of eHealth literacy and lower psychological symptoms [28] and higher prevention behaviors [29] could be confirmed. To sum up, research indicates that eHealth literacy is associated with prevention behaviors, the acquisition of knowledge, and people’s ability to cope with diseases, which confirms eHealth literacy as an important construct in examining people’s health behavior.

To cope with information overload and use the information from the internet, Norman and Skinner [15] proposed a set of different competencies: skills to read, identify, and understand different information to distinguish helpful from less helpful or even false or harmful information. These competencies represent a sequential process of handling available information. In the first step, basic cognitive skills are needed to search for information regarding a certain topic. In a subsequent cognitive process, information available must be distinguished as helpful or less helpful in order to answer specific questions. These steps represent an elaborated cognitive information process rather than a heuristic one. The distinction of cognitive processes was formerly described within dual-process theories in psychological literature and confirmed in multiple studies [30-32]. Dual-process theories distinguish between fast cognitive processes, which describe heuristic and holistic approaches representing intuitive, implicit cognitions, and slow cognitive processes, which are analytic and rule-based and focus on explicit learning [33]. Slow cognitive processes run serially and require cognitive capacity to answer or address specific questions. In the context of eHealth literacy, the handling of health information from the internet clearly represents a serial process of subsequent cognitions that require different competencies building on each other.

### eHEALS: Translations of the Original eHEALS Questionnaire and its Limitations

Since its publication, the original eHEALS questionnaire has been translated into many languages, including Italian [34,35], Spanish [36], Dutch [37], Chinese [38], Serbian [39], Korean [40], Indonesian [41] and German [42]. However, some of these studies could not confirm the 1-factorial model as assumed by Norman and Skinner [16]. Looking at many different validation studies of the eHEALS questionnaire, a consistent factorial structure has not been verified; 1-factorial [16,37,43], 2-factorial [42,44,45], and 3-factorial models [46-48] have been identified in different validation studies and languages. These results indicate that the eHEALS questionnaire lacks consistent factorial structure.

The German version of the questionnaire validated by Soellner and colleagues [42] especially lacks methodological and content-related accuracy. They developed an initial instrument for assessing eHealth literacy for the German-speaking community (G-eHEALS). However, Soellner and colleagues [42] did not meet scientific criteria substantially; first, they did not meet the criteria scientifically recommended for translation of instruments. Second, in their 2-factorial model content validity was questionable because some items reflected the subdimension of information appraisal rather than the assigned subdimension of information seeking (“I know how to use the health information I find on the internet to help me” or “I feel confident in using information from the internet to make health decisions”). In addition, Soellner and colleagues [42] collected their data on a limited sample of 327 students aged 16 to 21 years at only one type of school (gymnasium: a German school type preparing for university attendance), and people of older age were not considered for validation. However, as people of older age may be less familiar using the internet [49-51] and eHealth literacy especially depicts a particular digital literacy, the model proposed by Soellner and colleagues [42] is possibly not valid for assessing eHealth literacy in older people. Moreover, the educational level of the participants could not be considered within their biased study sample. Juvalta and colleagues [52], who used the G-eHEALS, have also collected their data on a limited sample of young parents (88.5% female). In another German-speaking study, Reder and colleagues [53] have shown a 3-factorial structure for the G-eHEALS. However, only women participated in this study, which is a limited sample for examining the validity of the G-eHEALS. Inconsistent findings and methodological limitations of these studies indicate an unclear factorial structure of the G-eHEALS.

Another limitation of the original eHEALS questionnaire refers to insufficient representation of an elaborated cognitive information process. The original scale does not reflect the above-mentioned complexity of an information process in its entirety. Petrič and colleagues [54] focused on this limitation and developed an extended eHealth literacy scale (eHEALS-E). Creating a 20-item questionnaire, they found a 6-factorial structure. Despite this extension and other concepts and questionnaires [55-57], eHEALS is still the instrument most used for measuring eHealth literacy.

### Aims of This Study

In summary, the G-eHEALS validated by Soellner and colleagues [42] was a valuable first approach to the important topic of eHealth literacy, but it underlies significant methodological limitations and lacks in psychometric quality. Nevertheless, as eHealth literacy could be confirmed as an important construct of health-related outcomes, the possibility of assessing eHealth literacy is crucial for health care practitioners and researchers in understanding health competence in German-speaking people. In response to the practical and scientific demands and described limitations, we developed a new instrument for measuring eHealth literacy with 4 objectives:

- Extension of the existing questionnaire of Norman and Skinner [16] by 8 nonoverlapping items proposed by Petrič and colleagues [54]. By combining the questionnaires, a better representation of the construct of eHealth literacy regarding the cognitive processes of seeking, identifying, and evaluating health information should be achieved.
- German translation of the items according to common scientific recommendations [58,59] to ensure content validity.
- Validation of the revised GR-eHEALS at a convenience sample in terms of construct and criterion validity. We decided to collect data in a convenience sample to reach participants with varied socioeconomic backgrounds. Furthermore, our goal was not to limit the sample in order to develop a measurement model that is as generic as possible.
- To our knowledge, there is no study examining measurement invariance of eHealth literacy between gender, age, or educational level in a German sample. Nevertheless, the interpretation of statistical differences between different groups of people requires measurement invariance between these groups [60]. As eHealth literacy represents competencies that are important for people regardless of their sociodemographic status, its measurement should obviously be independent of these influencing variables.

All in all, we are pursuing the study goals to develop a revised and validated instrument for measuring eHealth literacy. Further, we sought to examine the measurement invariance of the instrument regarding relevant sociodemographic variables.

## Methods

### Development of the New Instrument

The revised eHealth Literacy Scale (GR-eHEALS) is based on the original items from the eHEALS [16] extended by adding items from the eHEALS-E questionnaire from Petrič and colleagues [54]. The translation was conducted following the guidelines proposed by Beaton and colleagues [58] and Guillemin and colleagues [59] for translation of academic literature to ensure content validity. Accordingly, in a first step, 2 of the authors translated the items into German and merged these translations into a first translation proposal. In the second step, this proposal was discussed within a systematic expert panel consisting of the 2 translators and 2 psychologists who are experts in the context of health care and eHealth. The resulting second proposal was translated back into English in the third step to confirm that the essential meaning of the items is consistent with the original items. In the fourth step, cognitive interviews were conducted to make sure that all items are easy to understand, do not include offensive speech, and do not discriminate for age or gender. Interviewees were aged 23 to 72 years and had different educational backgrounds. The resulting final version of the translated and extended version consisted of 16 items. The original items and the translated items are displayed in Multimedia Appendix 1. Items 1 to 8 are translated from the original eHEALS questionnaire from Norman & Skinner [16], and items 9 to 16 are translated from the questionnaire (eHEALS-E) from Petrič and colleagues [54]. All subsequent nominations of item numbers refer to the item numbers mentioned in Multimedia Appendix 1. To validate the GR-eHEALS, we performed a prestudy in which we aimed to check for any complications in answering the translated items and to conduct an item analysis The results of this analysis are displayed in Multimedia Appendix 2. As the prestudy showed solid item characteristics, the developed instrument was considered good fitting for the purpose of the main study.

### Study Design and Participants

The cross-sectional study was conducted via Unipark (Tivian XI GmbH), an online survey tool, between October and November 2020. The ethics committee of the Faculty of Medicine of the University of Duisburg–Essen reviewed and approved this study (20-9592-BO).

All data were collected anonymously. Participants for this study were recruited via personal and occupational networks and online social networks (Xing, Facebook, LinkedIn). In our analyses, only complete data sets were considered. From a total of 1634 participants, 524 have completed our questionnaire in full, which represents a completion rate of 32.1% and can be considered typical for an online survey [61]. We excluded cases in which participants took less than 5:34 minutes (5% percentile) or more than 25:45 minutes (95% percentile) to complete the survey. Furthermore, we excluded 1 participant for being under 18 years old. As only 1 person indicated gender as diverse, we excluded this case in order to perform the analysis of measurement invariance of gender. The resulting sample consisted of 470 respondents. The sample size is in accordance with recommendations for validation studies [62,63]. Answering the questionnaire took 11:32 (SD 4:24) minutes on average. All data supporting the conclusion of the study are included in Multimedia Appendix 3.

In the main study, it was our objective to validate the GR-eHEALS in a convenience sample to verify its convergent, discriminant, and criterion validity and test for measurement invariance.

We verified convergent validity by assuming a positive correlation between eHealth literacy and health literacy, which measures a similar construct but does not take the source of information into account. Furthermore, we assumed eHealth literacy to be positively interrelated with internet confidence and negatively associated with internet anxiety as eHealth literacy particularly focuses on the gathering of information from the internet.

To verify discriminant validity, we captured impulsivity and common personality traits assuming no significant interrelations. As eHealth literacy reflects competencies in dealing with health-related information [15] rather than a personality trait, there should be no content-related overlaps between eHealth literacy and personality traits.

Additionally, we considered the possible outcome variables mental and physical health status and life satisfaction to examine criterion validity. Criterion validity of an instrument describes the ability to prove relationships between the construct itself and possible outcomes [64]. Thus, we expected eHealth literacy to be associated with above mentioned health-related variables.

The survey included the following questionnaires (sample items presented below are translations). Most scales were assessed on 5-point Likert scales from 1=strongly disagree to 5=strongly agree. Exceptions are separately explained below. Scales contained inverted items that were recoded prior to statistical analyses.

### Measurements

#### Health Literacy

Participants rated their health literacy on 16 items from the Health Literacy Questionnaire from Röthlin and colleagues [65]. A sample item is “How easy/difficult is it to find information about therapies for diseases that affect you?” Health literacy was measured on a 2-point scale (easy/hard). Therefore, it is used as a sum-score indicating the extent of health literacy between 0 and 16 (mean 12.63 [SD 2.99]). Cronbach alpha of this scale was .79.

#### Impulsivity

We used the 8-item Impulsive Behavior–8 Scale from Kovaleva and colleagues [66] to measure impulsivity (eg, “Sometimes I spontaneously do things that I should not have done”). Cronbach alpha of this scale was .72 (mean 2.78 [SD 0.59]).

#### Personality Traits

Personality traits (extraversion, neuroticism, openness, conscientiousness, and agreeableness) were each assessed by 2 items from Rammstedt and colleagues [67]. A sample item for neuroticism is “I get nervous and insecure easily.” Extraversion (mean 3.30 [SD 1.04]), neuroticism (mean 3.08 [SD 0.97]), openness (mean 3.61 [SD 0.99]), conscientiousness (mean 3.59 [SD 0.75]), and agreeableness (mean 3.15 [SD 0.76]) had Cronbach alphas of .79, .66, .62, .38, and .19, respectively. Due to low reliabilities, conscientiousness and agreeableness were excluded from the following analyses.

#### Further Constructs

In addition, we asked for internet confidence (3 items; mean 3.74 [SD 0.72], Cronbach alpha .89), internet anxiety (3 items; mean 1.81 [SD 0.82], Cronbach alpha .81) and single items to measure physical (mean 7.37 [SD 1.58]) and mental health (mean 7.27 [SD 1.90]) on 11-point Likert scales from 0=very bad health to 10=very good health (all self-formulated), and life satisfaction at a 5-point Likert scale from 1=not satisfied at all to 5=totally satisfied (mean 3.76 [SD 0.83]) from Beierlein and colleagues [68].

Furthermore, sociodemographic variables (age, gender, marital status, educational level, financial situation, internet availability, and community size) were considered to make sure that the sample represents the population.

### Statistical Analysis

All data analyses were conducted using R (R Foundation for Statistical Computing), RStudio, and several packages.

Prior to conducting confirmatory factor analysis (CFA), we performed an exploratory factor analysis (EFA) to evaluate whether data were suitable for factor analysis. We used the Kaiser-Meyer-Olkin (KMO) and Bartlett test of sphericity for evaluation. Factor extraction was conducted using maximum likelihood estimation with Promax oblique rotation and number of factors were identified by scree plot inspection and Kaiser criterion (eigenvalue >1). Factor loadings ≥0.4 were considered as significant [69].

Subsequently, we performed consecutive CFA and compared fit indices and factor loadings to confirm the best-fitting model by considering the recommendations of Hu and Bentler [70] who assume to achieve a comparative fit index (CFI) and Tucker Lewis index (TLI) about 0.95 and root mean square error of approximation (RMSEA) and standardized root mean square residual (SRMR) about 0.06 and 0.08, respectively. We used the robust maximum likelihood estimator as our prestudy showed that items were slightly negative skewed, and a robust estimator is more likely to produce less biased model statistics than maximum likelihood estimator [71].

Two-tailed Pearson correlations were conducted considering a significance level of 5% to examine convergent, discriminant, and criterion validity.

We performed tests of measurement invariance on our final model to examine whether the measurement is reliable for both genders as well as 2 age groups and 3 groups of educational level. For this purpose, we performed consecutive multigroup CFA with progressively stricter model assumptions by fixing an increasing number of model parameters for each of 3 measurement invariance models.

Measurement invariance—as a prerequisite for the interpretation of mean differences—is verified by 3 consecutive steps with increasingly strict model assumptions for (1) the number of factors and the pattern of factor-indicator relationships (configural invariance), (2) factor loadings (metric invariance), and (3) intercepts of indicators (scalar invariance) [72]. These 3 steps assume that there are no differences between observed groups regarding these parameters, and interpretation of mean differences is valid when scalar invariance is confirmed [73]. Differences between groups should only be interpreted when measurement invariance is confirmed since otherwise differences between groups may occur due to the fact that an instrument does not measure equally between different groups [60,73,74].

We applied a cutoff criterion of a difference of CFI (ΔCFI) of 0.01 as it is proposed as appropriate to assume invariance between two models [75,76]. Thus, for evaluation of measurement invariance we considered the model fit indices and difference of CFI between compared models.

## Results

### Sample Characteristics

Mean age of participants was 37.16 (SD 13.4, min 18, max 82, median 33) years. Sample characteristics of all other sociodemographic variables are shown in Table 1.

**Table 1:** Summary of sample characteristics (n=470).

Characteristics		Values, n (%)
**Gender**		
	Female	332 (70.6)
	Male	138 (29.4)
**Marital status**		
	Married	161 (34.3)
	Not married, in partnership	183 (38.9)
	Single	115 (24.5)
	Other	11 (2.3)
**Educational level**		
	Lower secondary school	5 (1.1)
	Upper secondary school	24 (5.1)
	University entrance qualification	77 (16.4)
	Vocational training	91 (19.4)
	University degree	273 (58.1)
**Financial situation**		
	Very good	9 (1.9)
	Good	47 (10.0)
	Middling	114 (24.3)
	Bad	220 (46.8)
	Very bad	80 (17.0)
**Internet availability**		
	Always available	288 (61.3)
	Mostly available	177 (37.7)
	Occasionally available	5 (1.1)
	Not available	0 (0.0)
**Community size**		
	Big city (>100,000 inhabitants)	244 (51.9)
	Medium city (>20,000 inhabitants)	88 (18.7)
	Small city (>5000 inhabitants)	76 (16.2)
	Rural village (<5000 inhabitants)	62 (13.2)

### Exploratory Factor Analysis

KMO revealed a value of 0.92 and Bartlett test of sphericity was highly significant (*P*<.001), indicating that data were suitable for factor analysis. Empirical Kaiser criterion and scree plot implied a 2-factor model. Table 2 shows factor loadings of the 2 factors.

As item 14 did not significantly load on any of the 2 factors it was excluded from the following analysis. The remaining 15 items were considered in the CFA.

**Table 2:** Results of exploratory factor analysis.

Item no	Factor 1	Factor 2
1	0.88	–0.06
2	0.80	0.03
3	0.84	0.00
4	0.97	–0.06
5	0.49	0.37
6	0.10	0.62
7	0.03	0.70
8	0.28	0.49
9	0.00	0.78
10	–0.12	0.78
11	–0.11	0.75
12	–0.07	0.56
13	0.12	0.56
14	0.32	0.31
15	0.44	0.01
16	0.44	–0.09

### Confirmatory Factor Analysis

In model 1, 15 items were assigned on the 2 factors identified by the EFA. Based on the content meanings of the underlying items, factor 1 represents information seeking and factor 2 represents information appraisal. However, items 13, 5, and 15 did not fit the factor proposed by the EFA in terms of their content. Therefore, item 13 was reassigned to information seeking whereas items 5 and 15 were reassigned to information appraisal in model 2. For model 3, we removed 6 items due to low factor loadings (<0.65). Moreover, we excluded 1 more item to develop a parsimonious model resulting in a 2-factorial model with 4 items on each of the 2 factors. Table 3 shows the model fits of the 3 models.

CFI, TLI, and SRMR practically meet the criteria of a good model fit. RMSEA is slightly above the recommendations of Hu and Bentler [70]. Considering the recommendations, model 3 shows an acceptable-to-good model fit.

**Figure 1:**
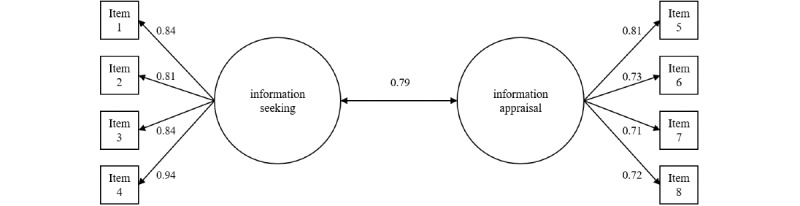
A 2-factorial, intercorrelated model of eHealth literacy.

Figure 1 depicts the structure of the 2-factorial model with its factor loadings. All item factor loadings were greater than λ=0.71.

Information seeking and information appraisal achieved satisfactory Cronbach alphas of .92 and .83, respectively. Table 4 shows the statistics of the final items. Based on mean and standard deviation, lower levels of information seeking and information appraisal are below a mean score of 2.99 and 3.20, respectively. Higher levels can be assumed above mean scores of 4.71 and 4.69, respectively.

**Table 3:** Results of the confirmatory factor analyses.

Model	Chi-square	df	CFI^a^	TLI^b^	RMSEA^c^	SRMR^d^	AIC^e^	BIC^f^
1	433.5	89	0.891	0.871	0.100	0.067	16029.832	16158.567
2	519.8	89	0.863	0.839	0.112	0.084	16136.608	16265.343
3	117.0	19	0.942	0.915	0.127	0.055	7782.043	7852.640

^a^CFI: comparative fit index.

^b^TLI: Tucker Lewis index.

^c^RMSEA: root mean square error of approximation.

^d^SRMR: standardized root mean square residual.

^e^AIC: Akaike information criterion.

^f^BIC: Bayesian information criterion.

**Table 4:** Descriptive statistics of the revised German eHealth Literacy Scale (GR-eHEALS) items.

Item			Mean (SD)		Median		Skew
**Information seeking**			3.85 (0.86)		4.00		–0.78
	1. Ich weiß, wie ich Internetseiten mit hilfreichen Gesundheitsinformationen finden kann.	3.93 (0.95)		4.00		–0.93	
	2. Ich weiß, wie ich das Internet nutzen kann, um Antworten auf meine Gesundheitsfragen zu erhalten.	4.04 (0.87)		4.00		–1.01	
	3. Ich weiß, welche Seiten mit Gesundheitsinformationen im Internet verfügbar sind.	3.63 (1.00)		4.00		–0.60	
	4. Ich weiß, wo ich im Internet hilfreiche Gesundheitsinformationen finden kann.	3.81 (1.01)		4.00		–0.89	
**Information appraisal**			3.95 (0.74)		4.00		–0.77
	5. Ich weiß Gesundheitsinformationen aus dem Internet so zu nutzen, dass sie mir weiterhelfen.	3.91 (0.88)		4.00		–0.77	
	6. Ich bin in der Lage, Internetseiten mit Gesundheitsinformationen kritisch zu bewerten.	4.18 (0.87)		4.00		–1.24	
	7. Ich kann zwischen vertrauenswürdigen und fragwürdigen Internetseiten mit Gesundheitsinformationen unterscheiden.	4.07 (0.84)		4.00		–0.93	
	8. Ich fühle mich sicher darin, Informationen aus dem Internet zu nutzen, um Entscheidungen in Bezug auf meine Gesundheit zu treffen.	3.62 (1.03)		4.00		–0.57	

### Validation of the GR-eHEALS

To examine convergent, discriminant, and criterion validity of the GR-eHEALS, we performed correlation analyses with the 2 factors (information seeking and information appraisal). Moreover, correlations of the 2 factors with demographic variables were calculated. Results are shown in Table 5. Both factors were strongly positively correlated with health literacy and internet confidence and strongly negatively correlated with internet anxiety. None of the 2 scales correlated significantly with impulsivity or extraversion. Information appraisal was interrelated with neuroticism while information seeking was associated with openness. Information appraisal was correlated with mental and physical health and life satisfaction, which was not true for information seeking. Furthermore, information seeking was significantly associated with age.

**Table 5:** Pearson correlation coefficients of the eHealth literacy factors.

Scales			Information seeking (*P* value)		Information appraisal (*P* value)
**Convergent validity**					
	Health literacy	0.43 (<.001)		0.53 (<.001)	
	Internet confidence	0.17 (<.001)		0.17 (<.001)	
	Internet anxiety	–0.21 (<.001)		–0.23 (<.001)	
**Discriminant validity**					
	Impulsivity	–0.06 (.16)		–0.05 (.28)	
	Extraversion	–0.03 (.58)		0.03 (.56)	
	Neuroticism	–0.08 (.09)		–0.14 (.001)	
	Openness	0.10 (.03)		0.07 (.12)	
**Criterion validity**					
	Mental health	0.06 (.20)		0.19 (<.001)	
	Physical health	0.06 (.21)		0.12 (.01)	
	Life satisfaction	–0.01 (.83)		0.12 (.01)	
**Sociodemographic variables**					
	Age	0.10 (.02)		0.06 (.16)	
	Gender	–0.03 (.55)		0.01 (.78)	
	Marital status	–0.02 (.71)		–0.07 (.15)	
	Educational level	–0.04 (.39)		–0.02 (.68)	
	Financial situation	–0.05 (.27)		0.04 (.45)	
	Internet availability	0.01 (.76)		0.02 (.71)	
	Community size	0.02 (.60)		–0.04 (.41)	

### Test of Measurement Invariance

Measurement invariance of the GR-eHEALS was performed to test whether the scale is a suitable measurement independently of gender, age, and educational level. Prior to these analyses, a median split was performed to separate participants into 2 groups according to age. Median age was 33 years. Also, to divide the study sample into 3 groups of educational levels, we separated participants into people who held a university degree, people who completed a vocational training, and people who had any school certificate. Results of the analyses are shown in Table 6.

Besides chi-square and fit indices, Table 6 shows the differences of CFI between models. Regarding measurement invariance of gender and education, all changes in CFI are below 0.01, indicating that model fits did not substantially decrease between more constraint models. Measurement invariance regarding age must be rejected as configural invariance could not be confirmed.

**Table 6:** Results of measurement invariance for gender, age, and education using multigroup confirmatory factor analysis.

Model		Chi-square	df	CFI^a^	TLI^b^	RMSEA^c^	SRMR^d^	ΔCFI^e^
**Gender^f^**								
	Configural^g^	154.937	38	0.94	0.905	0.135	0.056	0.006
	Metric	166.889	44	0.93	0.916	0.128	0.066	0.002
	Scalar	181.273	50	0.93	0.923	0.122	0.068	0.002
**Age^h^**								
	Configural^g^	187.672	38	0.92	0.883	0.150	0.059	0.021
	Metric	185.713	44	0.92	0.901	0.138	0.059	–0.002
	Scalar	197.419	50	0.92	0.913	0.130	0.060	0.001
**Education^i^**								
	Configural^g^	170.758	57	0.94	0.904	0.136	0.058	0.007
	Metric	174.474	69	0.94	0.926	0.119	0.061	–0.004
	Scalar	196.107	81	0.94	0.934	0.112	0.063	0.002

^a^CFI: comparative fit index.

^b^TLI: Tucker Lewis index.

^c^RMSEA: root mean square error of approximation.

^d^SRMR: standardized root mean square residual.

^e^Change in CFI compared to preceding model.

^f^Female n=332; male n=138.

^g^Change of CFI compared to model 3.

^h^Age>median n=240; age<median n=230.

^i^University degree n=273; vocational training n=91; school certificate n=106.

## Discussion

### Principal Findings

The results of our factor analyses show that eHealth literacy consists of 2 factors, information seeking and information appraisal. Our first study aim was to examine whether the measurement of eHealth literacy could be improved by adding nonoverlapping items from the eHEALS-E [54] to the original eHEALS [16]. We performed an EFA and several CFAs to examine the factorial structure of our instrument. Our analyses show that the measurement of eHealth literacy could not be improved by adding additional items to the well-established eHEALS questionnaire.

However, our study significantly contributes to the existing measurement of eHealth literacy. By strongly following scientific recommendations regarding academic translations, we developed the GR-eHEALS with high content validity. By taking statistical and content-related consideration into account when conducting factor analyses, we developed a measurement model of eHealth literacy with high content validity and acceptable-to-good model fit. Cronbach alpha was satisfactory for the 2 factors indicating good internal consistency and confirming reliability of the instrument.

Our findings on the examination of convergent, discriminant, and criterion validity of our instrument were not completely consistent with our expectations and require critical discussion. As expected, the 2 factors showed significant correlations with the convergent constructs of health literacy, internet confidence, and internet anxiety. By contrast, while impulsivity and extraversion consistently showed, as expected, no significant correlations with the 2 factors, neuroticism and openness indicated more inconsistent interrelations. Neuroticism was strongly negatively correlated with information appraisal, but not with information seeking. On the other hand, openness was only correlated with information seeking but not with information appraisal. To understand these unexpected correlational patterns, we examined findings of studies discovering the associations of personality traits and health-related constructs. Other studies showed that neuroticism is associated with lower health behavior self-efficacy and health behaviors [77] and lower internet use for learning and education [78]. These findings could indicate that neuroticism distorts cognitive processes of higher elaboration that are required for information appraisal but not necessarily for information seeking. Regarding the personality trait of openness, Bogg and Vo [79] have shown that people with higher openness more often search the internet regarding health-related topics. One could think that openness promotes people to search for new information in a sense of curiosity. However, the subsequent and cognitively demanding process of information appraisal may not be promoted by people’s openness.

Referring to the examination of criterion validity, positive correlations with the possible outcome variable mental health, physical health, and life satisfaction were expected, although only information appraisal was significantly related to these constructs. These results could be potentially explained by the idea that information seeking is a process that requires cognitive efforts but may not be sufficient to promote satisfaction and health status on its own but needs a high competency in information appraisal as a mandatory precondition. However, the search of information is a necessary process to perform the subsequent process of information appraisal.

To sum up, convergent validity of our instrument can be comprehensively confirmed. Examination of discriminant validity and criterion validity reveal unexpected findings that should be subjects of further studies. Despite our results not completely meeting our expectations, findings indicate that the 2 factors represent different cognitive processes in line with dual-process theories of analytic and rule-based processes: information seeking as a first of 2 consecutive competencies exclusively focuses on the process of searching information on the internet but not on a deeper application of the information found. Within a second consecutive competency built on information seeking, information appraisal describes a cognitive process of interpretation of information and its application on personal health-related questions.

Furthermore, we investigated the measurement invariance for gender, age, and educational level. The results of our study suggest that measurement invariance of the GR-eHEALS can be assumed for gender and educational level at a scalar level of invariance but not for age. Our study is the first to examine measurement invariance for these sociodemographic variables. Particularly regarding sample limitations of previous studies investigating eHealth literacy, the GR-eHEALS is the first instrument that can be deployed and interpreted regardless of gender and educational level. Therefore, future researchers are able to interpret statistical differences of these sociodemographic variables on eHealth literacy by using the GR-eHEALS. This is highly important as one could think of differential levels of eHealth literacy due to gender, which was confirmed for the construct of health literacy [80]. Regarding educational level, studies suggest that education also plays a role in the context of eHealth literacy [81,82], but, to our knowledge, neither used instruments confirmed to be measurement invariant.

Concerning the finding of inequality of our instrument with respect to age, one potential explanation could be that older people are less familiar with using the internet than younger people in terms of a digital divide [49] and have a different understanding of information seeking and information appraisal than younger people. Chesser and colleagues [83] suggest that age is a relevant variable in the context of eHealth literacy. Further, in our data we found significant interrelations of age and information seeking but not of age and information appraisal. This should be examined further in upcoming research.

In summary, prior research indicates that the investigation of differences of eHealth literacy of different groups of people is of high scientific interest. Nonetheless, previous studies were lacking considering statistical differences should not be interpreted unless measurement invariance is confirmed. With the GR-eHEALS, we close this gap and contribute substantially to the understanding of the concept of eHealth literacy and the interpretation of mean differences for gender and educational level.

Due to its high validity, the GR-eHEALS provides researchers and practitioners with a measurement for the increasingly important construct of eHealth literacy. As eHealth literacy is linked with many health-related outcomes and behaviors [19,26,27], the GR-eHEALS could provide a basis for educational programs to improve eHealth literacy by focusing on the main cognitive processes important for interpreting health information from the internet. Also, there is evidence that students lack in competencies regarding eHealth literacy [84]. Hence, the assessment and development of eHealth literacy should be a part of students’ curriculum to provide young people with the competencies needed to maintain or improve one’s health status. Consequently, the GR-eHEALS could be part of educational psychologists’ diagnostic repertoire as well as a foundation for specialist training programs in schools and universities. We propose that the results of the GR-eHEALS should be interpreted based on the 2 competencies for diagnostic and interventions of eHealth literacy considering the described mean scores for higher and lower levels of information seeking and information appraisal.

### Strengths and Limitations

The main strengths of this study are the high methodological and psychometric standards applied to develop GR-eHEALS and confirm its content, construct, and criterion validity. Furthermore, confirmation of measurement invariance is a state-of-the-art approach with strong practical implications regarding the interpretations of group differences.

One limitation of our study was that we measured eHealth literacy by self-assessment only. Since this construct is intended to measure skills and competencies, eHealth literacy should either be compared with actual behaviors or assessed using behavior-based measurement. Furthermore, our data were collected in a cross-sectional study. Therefore, correlational directions show relationships but are not interpretable causally. Future research should explore if our 2 factors show different effects on health-related outcomes. Additionally, as we used an online survey, participation by people familiar with the internet was more likely than by people who rarely use the internet. Thus, the possibility of selection bias should be considered. In our sample, a high proportion of people holding a university degree limits the representativeness regarding the education level. As in Germany about 19% of the population hold a university degree [85], our sample with a proportion of 58% holding a university degree clearly overrepresents academic persons. Even though it was our goal to collect data on a convenience sample, our study sample consisted of 71% female participants and cannot be considered as population-representative. Therefore, future studies should replicate our findings using a population-representative sample.

### Conclusion

eHealth literacy reflects the important competence of people in maintaining and improving their health status. This competence will become more and more important since the internet provides a rapidly increasing amount of health information with considerable bandwidth of quality and trustworthiness. The GR-eHEALS, with its 8 items on 2 factors, is a validated instrument to capture eHealth literacy in the German language. The GR-eHEALS contributes to the measurement of eHealth literacy in 3 ways: (1) instrument has high content validity because of a translation following scientific recommendations, (2) instrument has an acceptable-to-good model fit and confirms measurement invariance for gender and educational level, and (3) instrument revises the existing G-eHEALS and fills an important gap in measuring eHealth literacy to provide researchers and practitioners an accurate and valid assessment.

## Appendix

Multimedia Appendix 1 Original and translated items.

Multimedia Appendix 2 Item statistics of the prestudy (n=50).

Multimedia Appendix 3 Dataset.

## Abbreviations

CFA: confirmatory factor analysis
CFI: comparative fit index
EFA: exploratory factor analysis
eHEALS: eHealth Literacy Scale
eHEALS-E: extended eHealth Literacy Scale
G-eHEALS: German eHealth Literacy Scale
GR-eHEALS: revised German eHealth Literacy Scale
KMO: Kaiser-Meyer-Olkin test
RMSEA: root mean square error of approximation
SRMR: standardized root mean square residual
TLI: Tucker Lewis index
