# Supplementary material for: Measuring Electronic Health Literacy: Development, Validation, and Test of Measurement Invariance of a Revised German Version of the eHealth Literacy Scale
Source: J Med Internet Res. 2022 Feb 2;24(2):e28252. doi: 10.2196/28252 (PMC8851340; doi:10.2196/28252)
Supplement: Multimedia Appendix 1 [file jmir_v24i2e28252_app1.pdf]

Multimedia Appendix 1: Original and translated items

Item	Original item	Translated item
1	I know how to find helpful health resources on the Internet.	Ich weiß, wie ich Internetseiten mit hilfreichen Gesundheitsinformationen finden kann.
2	I know how to use the Internet to answer my health questions.	Ich weiß, wie ich das Internet nutzen kann, um Antworten auf meine Gesundheitsfragen zu erhalten.
3	I know what health resources are available on the Internet.	Ich weiß, welche Seiten mit Gesundheitsinformationen im Internet verfügbar sind.
4	I know where to find helpful health resources on the Internet.	Ich weiß, wo ich im Internet hilfreiche Gesundheitsinformationen finden kann.
5	I know how to use the health information I find on the Internet to help me.	Ich weiß Gesundheitsinformationen aus dem Internet so zu nutzen, dass sie mir weiterhelfen.
6	I have the skills I need to evaluate the health resources I find on the Internet.	Ich bin in der Lage, Internetseiten mit Gesundheitsinformationen kritisch zu bewerten.
7	I can tell high-quality from low-quality health resources on the Internet.	Ich kann zwischen vertrauenswürdigen und fragwürdigen Internetseiten mit Gesundheitsinformationen unterscheiden.
8	I feel confident in using information from the Internet to make health decisions.	Ich fühle mich sicher darin, Informationen aus dem Internet zu nutzen, um Entscheidungen in Bezug auf meine Gesundheit zu treffen.
9	I can easily extract the essential meaning of some health information on the Internet.	Es fällt mir leicht, die wesentliche Bedeutung von Gesundheitsinformationen aus dem Internet herauszufiltern.
10	Considering all health information on the Internet, I sometimes find it difficult to select the most relevant for my health.	Ich finde es schwierig, im Internet die Gesundheitsinformationen auszuwählen, die für mich wichtig sind.
11	The huge quantity of health information available on the Internet usually confuses me.	Die große Menge an Gesundheitsinformationen im Internet verunsichert mich.
12	I do not have any difficulties understanding the terminology used by some online health resources.	Ich habe Schwierigkeiten damit, die Fachbegriffe der Gesundheitsinformationen im Internet zu verstehen.
13	Sometimes, when I am confronted with a health issue, I am not sure where to start searching for information on the Internet.	Wenn ich eine Frage in Bezug auf meine Gesundheit habe, bin ich unsicher, wo ich die Suche nach Informationen im Internet beginnen soll.
14	Usually, I do not find helpful health information on the Internet.	Normalerweise finde ich keine hilfreichen Gesundheitsinformationen im Internet.

15	The Internet helps me to make decisions about my health more easily.	Das Internet hilft mir, Entscheidungen in Bezug auf meine Gesundheit zu treffen.
16	It is important for me to be able to access health-related online information.	Mir ist es wichtig, im Internet Zugriff auf gesundheitsbezogene Informationen zu haben.

Original items 1-8 come from the eHeals questionnaire from Norman & Skinner [16]
Original items 9-16 come from the eHeals-E questionnaire from Petrič and colleagues [54]
