# Supplementary material for: Measuring Electronic Health Literacy: Development, Validation, and Test of Measurement Invariance of a Revised German Version of the eHealth Literacy Scale
Source: J Med Internet Res. 2022 Feb 2;24(2):e28252. doi: 10.2196/28252 (PMC8851340; doi:10.2196/28252)
Supplement: Multimedia Appendix 2 [file jmir_v24i2e28252_app2.pdf]

Multimedia Appendix 2: Item statistics of the pre-study (N=50)

No	Item	Mean	Median	SD	Skew
1	Ich weiß, wie ich Internetseiten mit hilfreichen Gesundheitsinformationen finden kann.	3.62	4	0.92	-0.61
2	Ich weiß, wie ich das Internet nutzen kann, um Antworten auf meine Gesundheitsfragen zu erhalten.	3.80	4	0.81	-0.82
3	Ich weiß, welche Seiten mit Gesundheitsinformationen im Internet verfügbar sind.	3.08	3	1.07	-0.27
4	Ich weiß, wo ich im Internet hilfreiche Gesundheitsinformationen finden kann.	3.40	4	1.05	-0.66
5	Ich weiß Gesundheitsinformationen aus dem Internet so zu nutzen, dass sie mir weiterhelfen.	3.60	4	0.93	-0.55
6	Ich bin in der Lage, Internetseiten mit Gesundheitsinformationen kritisch zu bewerten.	3.82	4	1.06	-1.11
7	Ich kann zwischen vertrauenswürdigen und fragwürdigen Internetseiten mit Gesundheitsinformationen unterscheiden.	3.80	4	0.88	-0.71
8	Ich fühle mich sicher darin, Informationen aus dem Internet zu nutzen, um Entscheidungen in Bezug auf meine Gesundheit zu treffen.	3.50	4	1.02	-0.67
9	Es fällt mir leicht, die wesentliche Bedeutung von Gesundheitsinformationen aus dem Internet herauszufiltern.	3.48	4	0.89	-0.21
10	Ich finde es schwierig, im Internet die Gesundheitsinformationen auszuwählen, die für mich wichtig sind.	3.42	4	0.91	-0.44
11	Die große Menge an Gesundheitsinformationen im Internet verunsichert mich.	3.10	3	1.20	0.02
12	Ich habe Schwierigkeiten damit, die Fachbegriffe der Gesundheitsinformationen im Internet zu verstehen.	3.68	4	1.15	-0.84
13	Wenn ich eine Frage in Bezug auf meine Gesundheit habe, bin ich unsicher, wo ich die Suche nach Informationen im Internet beginnen soll.	3.76	4	1.17	-0.70
14	Normalerweise finde ich keine hilfreichen Gesundheitsinformationen im Internet.	3.68	4	0.98	-0.53
15	Das Internet hilft mir, Entscheidungen in Bezug auf meine Gesundheit zu treffen.	3.06	3	1.04	-0.12
16	Mir ist es wichtig, im Internet Zugriff auf gesundheitsbezogene Informationen zu haben.	3.92	4	0.94	-1.05
